# Multi-dimensional poverty and maternal care utilization in Nigeria

**DOI:** 10.3389/fpubh.2026.1853997

**Published:** 2026-08-11

**Authors:** Rolle Remi Ahuru

**Affiliations:** Department of Economics and Development Studies, Federal University, Otuoke, Bayelsa, Nigeria

**Keywords:** demographic and health survey, maternal care, Nigeria, poverty, women

## Abstract

**Introduction:**

This study examined the influence of Multi-Dimensional Poverty Index (MPI) on the utilization of maternal healthcare services in Nigeria.

**Methods:**

Secondary data from the 2023-24 Nigeria Demographic and Health Survey (NDHS) were analyzed. Two key indicators of maternal healthcare utilization were considered: [1] a minimum of eight antenatal care (ANC) visits, and [2] place of delivery (POD). Binary logistic regression was employed to identify the predictors of at least eight ANC visits and institutional delivery.

**Results:**

The result showed that 38.1% of the sampled women were multi-dimensionally poor. Additionally, 19% of women reported having at least eight ANC visits during pregnancy, while 42.5% delivered their most recent child in a health facility. The multivariate analysis showed that women who were multi-dimensionally poor were significantly less likely to attend ANC up to eight times and to deliver in a health facility.

**Conclusion:**

These results underscore the importance of addressing socioeconomic inequalities among Nigerian households to improve maternal healthcare utilization. Hence, multi-pronged approach should be adopted to improve household socioeconomic conditions in Nigeria.

## 1 Introduction

Pregnancy and childbirth are periods of heightened risk for women and their unborn babies (1). In 2020, it was estimated that approximately 800 women die daily from pregnancy-related complications, with about 95% of these deaths occurring in developing countries, particularly in sub-Saharan Africa (SSA) and South Asia (2). Antenatal care (ANC) has been identified as a key strategy for ensuring the well-being of mothers and their unborn babies (3). Through ANC, healthcare providers can detect potential risks early and initiate interventions to prevent complications (4).

The World Health Organization (WHO) recommends the Focused Antenatal Care (FANC) model, which prescribes a minimum of four ANC visits during pregnancy. The first visit should occur within the first 12 weeks of conception, the second at 16 weeks, the third at 20 weeks, and the fourth at 24 weeks of gestation (5). Evidence indicates that a higher frequency of ANC visits is associated with improved pregnancy outcomes and lower risks of stillbirth (6, 7). ANC services provided by skilled health professionals enable early detection of complications, timely treatment, disease prevention, routine immunization, micronutrient supplementation, and health education (8). In November 2016, WHO revised its recommendation, increasing the minimum number of ANC contacts from four to eight (9). Compared to four visits, attending ANC at least eight times can reduce perinatal mortality by up to 8 deaths per 1,000 births (8). Following the release of the WHO ANC guideline in 2016, Nigeria adopted the recommendation of a minimum of eight ANC contacts in 2017, replacing the previous focused ANC model of four visits (8, 9).

Despite these benefits, maternal care utilization remains low in SSA, where many women either do not attend ANC or give birth outside health facilities (10). For instance, the 2023–24 Nigeria Demographic and Health Survey (NDHS) reported that only 52% of pregnant Nigerian women attend at least four ANC visits during their most recent pregnancy, while just 43% delivered in health facilities (1, 11). These figures underscore the persistently low uptake of maternal healthcare in Nigeria, contributing to poor pregnancy outcomes (12, 13).

Maternal and neonatal mortality remain major public health challenges in Nigeria, where preventable maternal deaths continue to occur at alarming rates (4). In 2023, the country’s maternal mortality ratio (MMR) was estimated at 993 deaths per 100,000 live births, far above the Sustainable Development Goal (SDG) target of 70 per 100,000 live births (14). Nigeria records about 75,000 maternal deaths annually and accounts for roughly 35% of global maternal deaths (15, 16). These figures highlight the urgent need to understand the determinants of maternal healthcare utilization, particularly ANC and facility delivery, which are crucial for improving safe motherhood, human capital development, and national productivity (14).

Most existing studies on maternal healthcare utilization in Nigeria have focused on monetary measures of poverty, particularly household wealth quintiles (17). While numerous studies have explored socioeconomic and demographic predictors of maternal care use (4, 17), few have examined the role of multidimensional poverty. For instance, Xu et al. (1), Ekholuenetale et al. (8), and Oyedele (18) investigated household and community-level determinants of ANC utilization in Nigeria, and Xu et al. (1) applied multilevel modeling to examine predictors of facility delivery considering household wealth and community poverty levels. These studies generally found that poverty was negatively associated with maternal healthcare utilization (1, 2, 15).

However, evidence remains scarce on how multidimensional poverty, a measure that captures deprivations beyond income, affects maternal healthcare utilization in Nigeria. Compared with monetary measures, the Multidimensional Poverty Index (MPI) offers a broader understanding of deprivation. First, MPI reveals “hidden” poverty that may persist even in relatively affluent societies. Second, it recognizes that poverty is multifaceted, encompassing deprivations in health, education, and living standards (14). Third, it conceptualizes poverty beyond income, acknowledging that individuals may have financial resources yet still experience multiple forms of deprivation (19, 20).

Given these considerations, this study examines the impact of non-monetary (multidimensional) poverty on maternal healthcare utilization in Nigeria. Despite several government interventions to promote safe motherhood, utilization of maternal care services remains suboptimal. With poverty levels increasing, it is crucial to assess how multidimensional poverty influence access to essential maternal healthcare. Recent estimates show that about 63% of Nigerians are multidimensionally poor, deprived in health, education, and living standards (21). Poverty may hinder women’s ability to access modern maternal care due to high service costs and other barriers. Multidimensional poverty affects women’s healthcare usage through various pathways. Educational deprivation can limit health literacy and awareness of the benefits of ANC and skilled birth. Poor living conditions, inadequate transportation, and limited access to health facilities increase the costs and time of care seeking. Economic deprivation reduces women’s ability to afford healthcare services, while lack of access to information restricts exposure to health messages. Poverty is associated with low decision-making autonomy and social exclusion, which may limit access to healthcare. This study therefore investigates the influence of multidimensional poverty on the utilization of maternal healthcare services among Nigerian women.

## 2 Materials and methods

### 2.1 Data source

This study utilized secondary data from the 2023–24 NDHS, a nationally representative dataset comprising information on 39,050 women aged 15–49 years. The NDHS collected data using three standardized questionnaires: the Household Questionnaire, the Individual Women’s Questionnaire, and the Men’s Questionnaire. The 2023–24 survey adopted a two-stage stratified sampling design to ensure national representativeness. In the first stage, enumeration areas (EAs) were selected from the national sampling frame, while in the second stage, households were systematically selected within each EA.

The 2023–24 NDHS identified 74 sampling strata and listed 1,400 EAs, from which 30 households were selected per EA, yielding a total of 42,000 eligible women. Of these, 39,050 women completed the interview, resulting in a 99% response rate (2). The study recruited 8,006 women who had complete responses on all variables included in the analyses. The inclusion criteria for this study are having given birth to a child 2 years prior to the survey, and having complete responses for all the variables used for the analyses. The present study was restricted to data derived from the Individual Women’s Recode File (IR file), as it contains detailed information on women’s reproductive histories, maternal health, and socioeconomic characteristics. All analyses were weighted to account for the complex survey design and ensure representativeness, as recommended in the DHS analytical guidelines (14, 22).

### 2.2 Outcome variable

The study employed two outcome variables: (1) a minimum of eight ANC visits (≥8 ANC visits) and (2) place of delivery (POD). The first outcome variable, ≥8 ANC visits, aligns with the World Health Organization’s *revised ANC* model, which recommends that pregnant women should make at least eight visits during pregnancy (2, 9). This variable was coded as binary, where women who reported eight or more ANC visits were coded as 1, and those who reported less than ANC visits were coded as 0. The second outcome variable, place of delivery, indicates whether the most recent childbirth occurred in a health facility. This variable was also binary: women who delivered in a health facility (public or private) were coded as 1, while those who delivered outside a health facility (home or other locations) were coded as 0 (1).

### 2.3 Explanatory variables

#### 2.3.1 Multidimensional poverty index

The main explanatory variable in this study is the Multidimensional Poverty Index (MPI), a comprehensive measure that captures deficiencies in basic needs and essential human capabilities within households (14). The MPI extends beyond income-based measures by revealing diverse patterns of deprivation across different dimensions of well-being (UNDP and Oxford Poverty and Human Development Initiative (23).

Introduced in 2010 by the United Nations Development Programme (UNDP) and Oxford Poverty and Human Development Initiatives (OPHI), the MPI conceptualizes poverty as a multidimensional experience characterized by deprivations in health, education, and living standards. It reflects a lack of freedom and capability to lead a life of dignity and value, rather than merely a lack of income (24).

The MPI incorporates 10 indicators across the three core dimensions of human development, which are health, education, and living standards, and they are equally weighted. Within each dimension, the indicators are assigned weights: health and education indicators each have a weight of 1/6; while living standard indicators each have a weight of 1/18. For every household, the weighted sum of indicators in which deprivation occurs is calculated. Table 1 shows the variables used in computing multidimensional poverty.

**Table 1:** Variables used in the construction of multidimensional poverty index.

Aspects of deprivation	Indicators	Definitions of deprivations
Health (1/3)	Nutrition (1/6)	A household is deprived if it has at least one child that is underweight, stunted or wasted.
	Child mortality (1/6)	A household is deprived if it has at least one under-five child that died in the last 5 years.
Education (1/3)	Years of schooling (1/6)	A household is deprived if at least one of its members has less than 8 years of schooling.
	School attendance (1/6)	A household is deprived if it has at least one child aged 6–14 years that is out of school.
Living standards (1/3)	Electricity (1/18)	A household is deprived if it lacks access to electricity.
	Cooking fuel (1/18)	A household is deprived if rubbish is disposed off on a public heap, burned, disposed off by other means or it lack waste disposal.
	Housing (1/18)	A household is deprived if at least two of the following dwelling structural components are of poor quality: Walls (grass, mud, compacted earth, unified mud bricks, wood, iron sheets or other materials. Roofs (grass, plastic sheeting or other materials) Floor (sand, smoothed mud, wood or other materials).
	Assets (1/18)	Members of the household are considered deprived in assets if it does not own more than one of radio, TV, Telephone, bike, motorbike, refrigerators, computer or animal cart.
	Drinking water (1/18)	Households is considered deprived if its members do not have access to safe drinking water according to MDG guidelines or safe drinking water is at least 30 min’ walk from home round trip.
	Sanitation (1/18)	Members of the particular households are considered deprived if there are non-improved sanitation facilities, according to MDG guidelines.

A household is classified as multidimensionally poor if the weighted sum of its deprivations is at least 33% of the total possible deprivations. Households are considered severely poor if the deprivation score is 50% or higher, and extremely poor if it reaches 75% or higher (14, 24). In this study, the MPI was treated as a binary variable, with women from households that are multidimensionally poor coded 1, and those that are not from multidimensionally poor homes coded 0.

### 2.4 Control variables

Several sociodemographic and economic characteristics were included as control variables, all derived from the 2023–24 NDHS. They include maternal education, maternal age, place of residence, employment status, health insurance enrolment, region, and barriers to accessing healthcare. Maternal education measures the respondent’s highest level of education, and it is coded as “1” for non-formal education, “2” for primary education, “3” for secondary education and “4” for higher education. Maternal age measures the current age of the respondents, and it considered seven age categories:15–19, 20–24, 25–29, 30–34, 35–39, 40–44, and 45–49 years. These groups were coded in ascending order from 1 to 7. Place of residence identifies the current location of the residents and considers whether the respondent lives in urban or rural areas. We coded rural as “1” and urban as “2.” Employment status examines whether the respondent was working at the time of the survey. Women not working were coded “1,” while those working were coded “0.” Health insurance measures whether the respondent was enrolled in any health insurance scheme or not, enrolment into health insurance was coded “2,” while non enrolment coded “1.” Region considered which of the six geopolitical zones the respondents come from. Two broad categories were used based on socioeconomic similarities. Women from northern parts of the country were coded “1” while those from the Southern parts of the country were coded “2.” The NDHS assesses multiple constraints women may face in seeking healthcare, including needing permission, high costs, distance, and reluctance to go alone. Women who reported any one of these barriers were coded “1,” and those without any of these barriers were coded “2.”

### 2.5 Data analysis

Three analytical approaches were employed in this study. First, descriptive statistics were used to summarize the characteristics of the respondents. Means and proportions were computed, and bivariate analyses were conducted to examine the distribution of multidimensional poverty, at least eight ANC and facility delivery across the covariates. Second, binary logistic regression analysis was applied to identify the predictors of the two outcome variables (1) ≥8 ANC visits and (2) place of delivery (POD). The MPI was computed using the Foster–Alkire method developed by OPHI. Fourth, post-estimation diagnostics were used to assess estimated models. The Hosmer–Lemeshow goodness-of-fit test was employed to assess the goodness-of-fit of the logistic regression model, ensuring that the predicted probabilities did not significantly differ from the observed outcomes (25). Additionally, the Omnibus Test of Model Coefficients was used to determine whether the inclusion of the independent variables significantly improved the explanatory power of the model indicating that the explained variance is statistically greater than the unexplained variance, as supported by earlier studies (5).

## 3 Results

### 3.1 Descriptive statistics

In Table 2, the descriptive statistics for the variables is presented using mean values. The summary statistics of the variables included in this study are presented in Table 2. The analysis focused on 8,006 women who had given birth within the 5 years preceding the 2023–24 NDHS survey. Regarding place of residence, 40.5% of the respondents lived in urban areas, while the remainder resided in rural communities. Most of the women attained secondary education, and 36.7% were below 24 years of age. Additionally, 64.6% of the respondents were employed at the time of the survey. The majority of women were from Northern Nigeria (61.4%), and 52.6% reported facing barriers to accessing healthcare facilities.

**Table 2:** Descriptive statistics of the variables used.

Variables	%
Maternal age	
15–19	20.3%
20–24	16.4%
25–29	17.1%
30–34	14.3%
35–39	12.9%
40–44	9.7%
45–49	9.3%
Maternal education	
Non-formal	34.6%
Primary	15.2%
Secondary	39.8%
Higher	10.3%
Place of residence	
Rural	59.5%
Urban	40.5%
Employment status	
Not working	35.4%
Working	64.6%
Heath insurance enrolment	
Not enrolled	97.3%
Enrolled	2.7%
Region	
Southern	33.6%
Northern	61.4%
Barriers in accessing health facilities	
Facing one or more barriers	47.9%
Facing no barriers	52.0%

Source: calculated by author.

**Figure 1:**
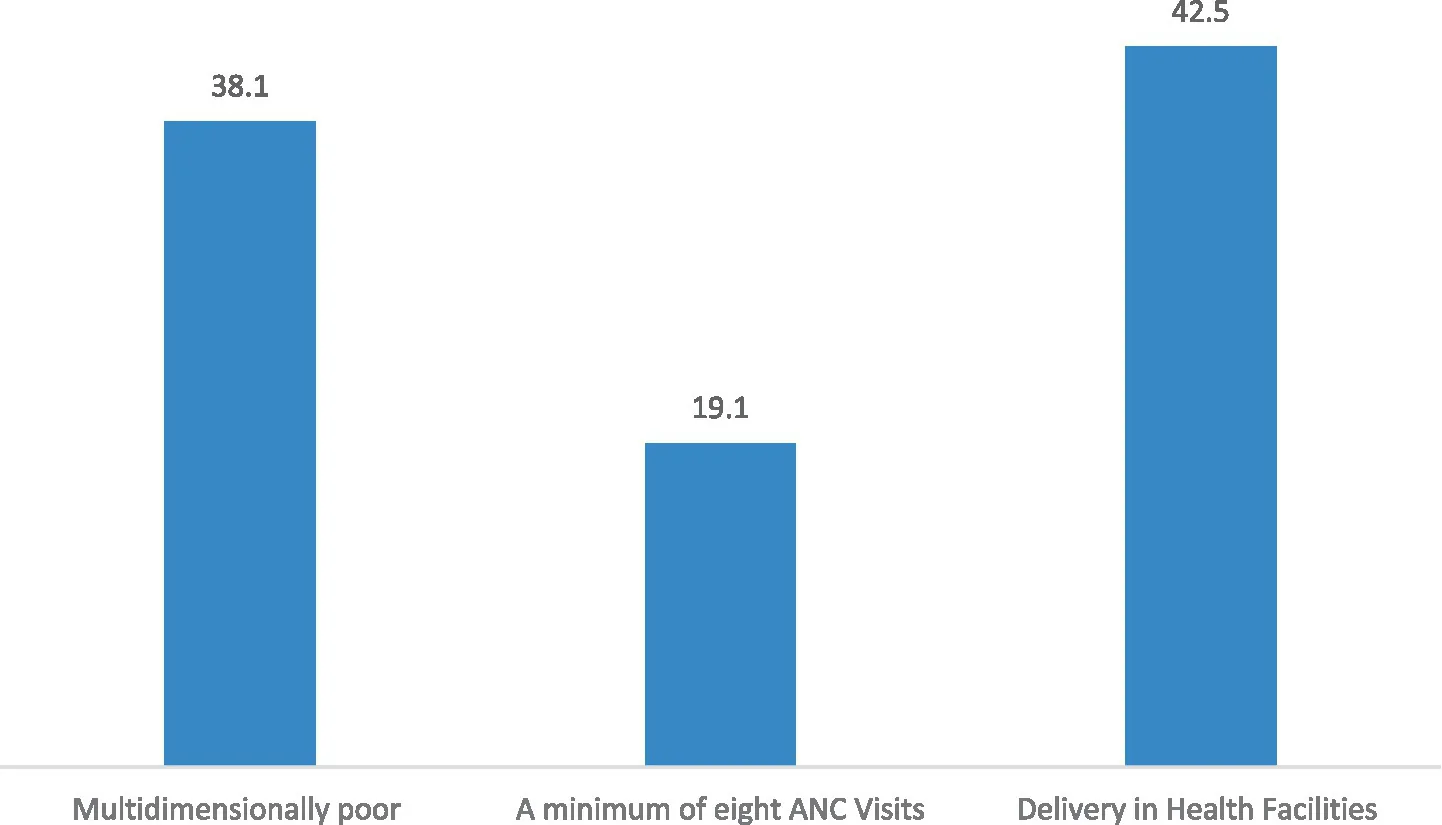
The proportion of women that are multidimensionally, undertake a minimum of eight ANC visits and had their childbirth in health facilities.

In Figure 1, the proportion of women that adhered to eight ANC contacts, delivered in health facilities and multidimensionally poor are presented. Results show that 38.1% of the sampled women were multidimensionally poor. Approximately 19.0% of women attend ANC up to eight times, while 42.5% delivered their most recent child in a health facility.

### 3.2 Bivariate analyses of ≥8 ANC visits in pregnancy

In Table 3, the factors associated with ≥8 ANC visits in Pregnancy were examined using Chi-square tests and set at 10% level of significance. The data shows that all the variables are statistically associated with a minimum of eight ANC contacts.

**Table 3:** Bivariate Analyses of ≥8 ANC visits and Other Covariates.

Variables	≥8 ANC visits			Total No. (*N* = 8,006)
	No (6,485)	Yes (1,521)	*p*-value	
	No. (%)	No. (%)		No. (%)
Multidimensional poor index (MPI)				
No	1,729 (56.7)	1,321 (43.3)	<0.001	3,050 (38.1)
Yes	4,756 (96.0)	200 (4.0)		4,956 (61.9)
Maternal age				
15–19	1,493 (91.9)	132 (8.1)	0.023	1,625 (20.3)
20–24	1,192 (90.8)	121 (9.2)		1,313 (16.4)
25–29	1,261 (92.1)	108 (7.9)		1,369 (17.1)
30–34	888 (77.6)	257 (22.4)		1,145 (14.3)
35–39	792 (76.6)	241 (23.3)		1,033 (12.9)
40–44	499 (64.2)	278 (35.8)		777 (9.7)
45–49	360 (48.4)	384 (51.5)		745 (9.3)
Maternal education				
Non-formal	2,722 (98.3)	48 (1.7)	0.012	2,770 (34.6)
Primary	1,044 (85.9)	172 (14.1)		1,216 (15.2)
Secondary	2,406 (75.5)	780 (24.5)		3,186 (39.8)
Higher	313 (37.5)	521 (62.5)		834 (10.3)
Place of residence				
Rural	2,921 (90.1)	321 (9.9)	<0.001	3,242 (59.5)
Urban	3,564 (74.8)	1200 (25.2)		4,764 (40.5)
Employment status				
Not working	2,390 (84.4)	445 (15.6)	<0.001	2,835 (35.4)
Working	4,095 (79.2)	1,076 (20.8)		5,171 (64.6)
Heath insurance enrolment				
No	6,396 (82.1)	1,394 (17.9)	0.002	7,790 (97.3)
Yes	89 (41.2)	127 (58.8)		216 (2.7)
Region				
Southern	1,701 (63.2)	989 (36.8)	0.015	2,690 (33.6)
Northern	4,784 (90.0)	532 (10.0)		5,316 (61.4)
Barriers in accessing health facilities				
Yes	3,593 (93.7)	241 (6.3)	0.017	3,834 (47.9)
No	2,892 (69.3)	1,280 (30.7)		4,172(52.1)

Source: calculated by author.

The chi-square analysis identified several factors that were significantly associated with attending at least eight ANC visits during pregnancy. The data in Table 3 shows that a higher proportion of women from non-multidimensionally poor households attended at least eight ANC visits compared to those from multidimensionally poor households (43.3% vs. 4.0%). With respect to maternal age, the highest proportion of women who attended at least eight ANC visits was observed among those aged 45–49 years, while the lowest proportion was among women aged 15–19 years. ANC attendance also increased with educational attainment. Only 1.7% of women with no formal education attended at least eight ANC visits, compared with 24.5 % of those with secondary education and 62.5% of those with higher education. Furthermore, a greater proportion of urban women attended the recommended minimum of eight ANC visits than rural women (25.2% % vs. 9.9%). Women enrolled in health insurance were slightly more likely to attend at least eight ANC visits than those who were not enrolled (58.8% vs. 17.9%). Regional differences were also observed, with a higher proportion of women in Southern Nigeria attending at least eight ANC visits compared with women in Northern Nigeria (36.8% vs. 10.0%). In addition, women who reported no barriers to accessing healthcare facilities were more likely to attend at least eight ANC visits than those who reported at least one barrier (30.7% vs. 6.3%).

### 3.3 Bivariate analyses of facility delivery

In Table 4, the factors associated with institutional delivery are examined using Chi-square tests and set at 10% level of significance.

**Table 4:** Bivariate analyses of institutional delivery and other Covariates.

Variables	Delivered in Health Institutions			Total No. (*N* = 8,006)
	No (4,603)	Yes (3,403)	*p*-value	
	No. (%)	No. (%)		No. (%)
Multidimensional poor index (MPI)				
No	860 (28.2)	2,190 (71.8)	0.020	3,050 (38.1)
Yes	3,743 (75.5)	1,213 (24.5)		4,956 (61.9)
Maternal age				
15–19	1,230 (75.7)	395 (24.3)	0.045	1,625 (20.3)
20–24	1,076 (81.9)	237 (18.1)		1,313 (16.4)
25–29	801 (58.5)	568 (41.5)		1,369 (17.1)
30–34	600 (52.4)	545 (47.6)		1,145 (14.3)
35–39	443 (42.9)	590 (57.1)		1,033 (12.9)
40–44	277 (35.6)	500 (64.4)		777 (9.7)
45–49	176 (23.6)	568 (76.2)		744 (9.3)
Maternal education				
Non-formal	2,327 (84.1)	443 (15.9)	<0.001	2,770 (34.6)
Primary	790 (64.9)	426 (35.0)		1,216 (15.2)
Secondary	1,352 (42.4)	1,834 (57.6)		3,186 (39.8)
Higher	134 (16.1)	700 (83.9)		834 (10.3)
Place of residence				
Rural	2,226 (68.5)	1,024 (31.5)	<0.001	3,250 (40.5)
Urban	2,378 (50.0)	2,378 (50.0)		4,756 (59.5)
Employment status				
Not working	2,379 (83.9)	456 (16.1)	0.023	2,835 (35.4)
Working	2,224 (58.0)	2,947 (42.0)		5,171 (64.6)
Heath insurance enrolment				
No	4,510 (57.9)	3,280 (42.1)		7,790 (97.3)
Yes	93 (43.0)	123 (57.0)		216 (2.7)
Region				
Southern	995 (37.0)	1,695 (63.0)	0.021	2,690 (33.6)
Northern	3,608 (67.8)	1,708 (32.2)		5,316 (61.4)
Barriers in accessing health facilities				
Yes	2,517 (65.4)	1,317 (34.4)	0.031	3,834 (47.9)
No	2,086 (50.0)	2,086 (50.0)		4,172 (52.1)

Source: calculated by author.

The data showed that all the variables are statistically associated with facility delivery. The chi-square analysis identified several factors that were significantly associated with institutional delivery. A higher proportion of women from non-multidimensionally poor households delivered in health institutions compared to those from multidimensionally poor households (71.8% vs. 24.5%). Regarding maternal age, the highest proportion of institutional deliveries was observed among women aged 45–49 years (76.2%), while the lowest proportion was among those aged 20–24 years (18.1%). Institutional delivery also increased with educational attainment, with only 15.9% of women with no formal education delivering in health facilities compared to 83.9% of women with higher education. Furthermore, a greater proportion of urban women delivered in health institutions than rural women (50.0% vs. 31.5%). Similarly, women enrolled in health insurance were more likely to deliver in health facilities than those without health insurance coverage (57.0% vs. 42.1%). Regional differences were also evident, as a higher proportion of women residing in Southern Nigeria had institutional deliveries compared to their counterparts in Northern Nigeria (63.0% vs. 32.2%). In addition, women who reported no barriers to accessing healthcare facilities were more likely to deliver in health institutions than those who experienced at least one barrier (50.0% vs. 34.4%).

### 3.4 Logistic regression results on the impact of multidimensional poverty on ≥8 ANC visits

The results of the logistic regression analysis for the determinants of ≥8 ANC visits are presented in Table 5, highlighting several factors that significantly influence ANC utilization among Nigerian women. The findings indicate that women living in multidimensional poverty were less likely to attend at least eight ANC visits (aOR = 0.273, 95% CI: 0.236–0.298). In reference to women aged (15–19) years, those aged (40–44) years (aOR = 2.786, 95% CI: 2.568–3.123) and (45–49) years (aOR = 5.897, 95% CI: 3.098–6.098) were more likely to attend at least eight ANC visits.

**Table 5:** Logit model estimation of the determinants of ≥8 ANC visit.

Variables	Adjusted odds ratio	*p*-value	95% confidence interval
Multidimensional poor index (MPI)			
No (Ref)	1.00	–	–
Yes	0.273	0.002	0.236–0.298
Maternal age			
15–19 (Ref)	1.00	–	–
20–24	2.098	0.234	1.998–2.711
25–29	2.099	0.325	2.011–2.356
30–34	3.011	0.434	2.986–3.071
35–39	3.078	0.986	2.986–3.088
40–44	2.786	0.003	2.568–3.123
45–49	5.897	0.001	3.098–6.098
Maternal education			
Non-formal (Ref)	1.00	–	–
Primary	0.986	0.386	0.211–1.098
Secondary	3.864	0.043	2.986–3.988
Higher	4.086	0.012	3.086–4.099
Place of residence			
Rural (Ref)	1.00	–	–
Urban	2.965	0.098	2.876–3.065
Employment status			
Not employed (Ref)	1.00	–	–
Employed	2.986	0.021	2.767–2.998
Heath insurance enrolment			
Not enrolled (Ref)	1.00	–	–
Enrolled	2.883	0.002	2.798–3.034
Region			
Northern (Ref)	1.00	–	–
Southern	3.128	0.004	3.115–3.211
Barriers in accessing health facilities			
No (Ref)	1.00	–	–
Yes	2.689	0.003	1.084–2.876
Mean dependent Variable	0.19	SD dependent variable	0.200
Hosmer–Lemeshow	37.63 (0.79)	Number of observations	8,006
Omnibus test	3,442.58 (0.00)*		

Ref: reference category. Source: calculated by author.

Educational attainment emerged as an important determinant. Furthermore, comparing to non-formal education, women with secondary education (aOR = 3.864, 95% CI:2.986–3.988) and higher education (aOR = 4.086, 95% CI: 3.086–4.099) were significantly more likely to attend at least eight ANC visits.

Employment status also played a significant role. Employed women (aOR = 2.986, 95% CI: 2.767–2.998) were more likely to attend at least eight ANC visits. In addition, health insurance coverage was a strong positive predictor, as insured women (aOR = 2.883, 95% CI: 2.798–3.034) were more likely to attend at least eight ANC visits. Also, place of residence influence uptake of maternal care such that women who reside in urban parts (aOR = 2.965, 95% CI: 2.876–3.065) were significantly more likely to attend at least eight ANC visits compared to those in rural parts of the country.

Regional differences were also evident: women from Southern Nigeria were more likely to attend at least eight ANC visits than those from the North (aOR = 3.128, 95% CI: 3.115–3.211). Finally, women who reported no barriers to accessing health facilities were significantly more likely to attend at least eight ANC visits in pregnancy (aOR = 2.689, 95% CI: 0.054–2.876).

The logistic regression model was statistically significant, as indicated by the Omnibus Test of Model Coefficients (*χ*^2^ = 3,442, *p* < 0.001), showing that the predictors jointly improved prediction of the outcome variable. Furthermore, the Hosmer–Lemeshow goodness-of-fit test was non-significant (*χ*^2^ = 37.63, *p* = 0.79), suggesting that the model fit the data adequately. Therefore, the model is both statistically significant and demonstrates acceptable goodness of fit.

### 3.5 Logistic regression results on the impact of multidimensional poverty on facility delivery

The logistic regression results for the place of delivery are presented in Table 6. A significant association exists between multidimensional poverty and place of delivery, indicating that women living in multidimensional poverty were less likely to deliver their children in health facilities (aOR = 0.827, 95% CI: 0.822–0.829). Age was positively associated with health facility delivery. In reference to women aged (15–19) years, those aged (40–44) years (aOR = 1.259, 95% CI: 1.209–1.262) and (45–49) years (aOR = 1.987, 95% CI: 1.098–2.112) were significantly more likely to have their child births in health facilities.

**Table 6:** Logit model estimation of the determinants of place of delivery.

Variables	Adjusted odds ratio	*p*-value	95% confidence interval
Multidimensional poor index (MPI)			
No (Ref)	1.00	–	–
Yes	0.827	0.034	0.822–0.829
Maternal age			
15–19 (Ref)	1.0	–	–
20–24	0.987	0.214	0.678–1.236
25–29	1.238	0.345	0.987–1.678
30–34	2.986	0.656	1.986–2.998
35–39	1.786	0.487	0.568–1.865
40–44	1.259	0.091	1.209–1.262
45–49	1.987	0.021	1.098–2.112
Maternal education			
Non-formal (Ref)			
Primary	0.998	0.865	0.867–1.278
Secondary	1.589	0.045	1.234–1.897
Higher	1.716	0.034	1.632–1.744
Place of residence			
Rural (Ref)	1.0	–	–
Urban	1.046	0.001	1.033–1.050
Employment status			
Not working (Ref)	2.380	0.045	1.082–3.093
Working	1.916	0.098	1.804–1.970
Heath insurance enrolment			
Not enrolled (Ref)	1.00	–	–
Enrolled	1.735	0.043	1.034–1.840
Region			
Northern (Ref)	1.00	–	–
Southern	2.489	0.021	1.987–2.556
Barriers in accessing health facilities			
No (Ref)	1.00	–	–
Yes	3.876	0.002	2.896–3.986
ANC visits in pregnancy			
No (Ref)	1.00	–	–
Yes	2.98	0.023	2.86–3.01
Mean dependent variable	0.425	SD dependent variable	0.500
Hosmer–Lemeshow	41.43 (0.89)	Number of observations	8,006
Omnibus test	4,442.78 (0.00)		

Source: Calculated by author. Ref: reference category.

Maternal education also exerted a strong positive influence on institutional delivery. In reference to women who had non-formal education, those who had secondary (aOR = 1.589, 95% CI: 1.234–1.897) and higher education (aOR = 1.716, 95% CI: 1.632–1.744) were significantly more likely to have their child births in health facilities.

Similarly, urban residence significantly improved the likelihood of health facility delivery (aOR= 1.046, 95% CI: 1.033–1.050), while employment status was another positive predictor, hence, employed women were more likely to deliver in health facilities compared to unemployed women (aOR = 2.380, 95% CI: 1.082–3.093).

Health insurance coverage was associated with increased use of facility-based delivery (aOR = 1.735, 95% CI: 1.034–1.840). Regional variation was also significant, as women from Southern Nigeria were more likely to deliver in health facilities than those from the North (aOR = 2.489, 95% CI: 1.987–2.556). Finally, women who reported no barriers to accessing healthcare facilities were significantly more likely to deliver in health facilities (aOR= 3.876, 95% CI: 2.896–3.986). In reference to women who did not utilize ANC in pregnancy, those who did (aOR = 2.98, 95% CI:2.86–3.01) were significantly more likely to deliver their babies in health institutions.

The logistic regression model was statistically significant according to the Omnibus Test of Model Coefficients (*χ*^2^ = 4,442, *p* < 0.001), indicating that the independent variables jointly contributed to predicting the outcome variable. The Hosmer–Lemeshow goodness-of-fit test was not significant (*χ*^2^ = 41.43, *p* = 0.89), suggesting that the model fit the data adequately. Therefore, the model provides a good fit to the data and has significant predictive power.

## 4 Discussion of findings

The primary objective of this study was to evaluate the association between the MPI and maternal care utilization, specifically, attending at least eight ANC visits in pregnancy and health facility delivery, with special focus on the individual and household factors associated with variations in maternal care use.

The findings underscore the importance of addressing socioeconomic disparities between the poor and non-poor, as reflected in the inverse association between MPI and maternal care utilization. Women living in multidimensional poverty were significantly less likely to attend at least eight ANC visits in pregnancy and deliver in health facilities. This pattern is consistent with findings from previous studies in Nigeria and other developing contexts (1, 8, 14, 26), reinforcing the need for targeted interventions that promote equitable access to maternal health services among disadvantaged populations.

Approximately 38.1% of women in the sample were identified as multidimensionally poor, highlighting the high prevalence of deprivation among women of reproductive age in Nigeria. This figure is comparable to findings from Malawi, where 34% of women were reported to experience multidimensional poverty (14). Furthermore, only 19% of women attended at least eight ANC visits in pregnancy, and 42.5% delivered their most recent child in a health facility, these figures remain below the WHO’s global recommendations (16). ANC provides an opportunity for healthcare practitioners to screen for complications and supports maternal and fetal well-being, while facility-based delivery remains a key component of safe motherhood (1, 12). These findings highlight the need for maternal health intervention programs aimed at expanding ANC and facility delivery coverage across Nigeria.

The results indicate that older women were more likely to utilize maternal care, both in terms of attending at least eight ANC visits and facility-based deliveries. This association may reflect the theoretical insights of Grossman’s (27) health capital model, which suggests that individuals possess a stock of health that diminishes over time and may require investment to maintain. Older women may, therefore, be more inclined to perceive pregnancy care as an important health investment (28, 29). These findings suggest the importance of interventions that encourage younger women, who were generally less likely to utilize maternal care services, to engage more actively in pregnancy-related healthcare.

The analysis further shows clear disparities in maternal care utilization between urban and rural women. Urban women were significantly more likely to attend at least eight ANC visits in pregnancy and deliver in health facilities. These findings are consistent with previous Nigerian studies (1, 5, 17). The observed differences may be related to characteristics commonly associated with urban areas, including closer proximity to healthcare facilities, greater employment opportunities, higher literacy levels, and increased exposure to health information through mass media (2, 8). Consequently, rural-focused interventions, such as mobile clinics, improved rural health infrastructure, and efforts to address geographic and socioeconomic barriers, remain important for reducing urban–rural disparities in maternal healthcare utilization.

Consistent with global evidence, maternal education was positively associated with both ANC attendance and facility delivery. Educated women were more likely to exhibit higher health literacy, greater decision-making autonomy, and increased awareness of maternal health services. Grossman’s (27) framework also suggests that educated people are more efficient in health production and promotion. Therefore, policies that expand women’s educational opportunities, including scholarships, affirmative admission criteria, and gender-targeted educational programs, may be associated with improvements in maternal health service utilization in Nigeria (17).

Significant regional disparities were observed, with women in Southern Nigeria being more likely to access both ANC and health facility delivery than their Northern counterparts. This finding aligns with past related studies (2, 4, 5) and may be related to religious, cultural, and structural differences across regions. In Northern Nigeria, cultural practices rooted in Islamic traditions, such as purdah (female seclusion), have been reported to be associated with restrictions on women’s mobility and healthcare-seeking autonomy (30, 31). In addition, educational attainment, women’s autonomy, and the distribution of health facilities tend to be lower in the Northern region (11). These findings highlight the importance of region-specific interventions, including community sensitization, gender empowerment initiatives, and equitable resource allocation, in efforts to reduce maternal health inequalities.

The study also reveals a strong positive association between health insurance coverage and maternal care utilization. Women enrolled in health insurance schemes were significantly more likely to attend at least eight ANC visits in pregnancy and deliver in health facilities. These results are consistent with findings from Nigeria (2, 17), Ethiopia (32, 33), Ghana (34), and other low- and middle-income countries (35). Health insurance coverage was associated with fewer financial barriers and greater utilization of skilled maternal care services. These findings suggest that expanding health insurance coverage may support Nigeria’s progress toward Sustainable Development Goal (SDG) 3, particularly targets 3.1 (reducing maternal mortality), 3.2 (reducing child and neonatal mortality), 3.7 (improving sexual and reproductive health), and 3.8 (achieving universal health coverage) (21).

The study found that women who attended ANC had higher odds of delivering in a health facility than those who did not attend ANC. This finding is consistent with previous studies conducted in Nigeria (36, 37) and other countries in SSA (38), which reported that ANC attendance is a significant predictor of institutional delivery. The observed association suggests that ANC attendance promotes facility-based delivery by increasing women’s contact with healthcare providers, improving their knowledge of pregnancy and childbirth, and enhancing birth preparedness and complication readiness. During ANC visits, women receive health education on the importance of skilled birth attendance, are screened for obstetric risks, develop individualized birth preparedness plans, and are referred to appropriate health facilities when necessary. Consequently, women who attend ANC are more likely to choose institutional delivery than those with limited or no ANC attendance. Overall, these findings underscore the importance of promoting adequate ANC utilization as a strategy for increasing institutional delivery and reducing maternal and neonatal morbidity and mortality.

## 5 Limitations

Despite the robustness of the findings, several limitations must be acknowledged.

First, while key covariates were controlled for, the study may not have accounted for all unobservable confounders such as local health policies or cultural dynamics that could influence maternal care use. Second, the cross-sectional nature of the data limits causal inference, as only associations, not temporal effects, can be inferred. Third, the absence of information on the quality of maternal care services constrains a more nuanced understanding of access versus quality. Future research should employ longitudinal data and incorporate quality-of-care indicators to provide deeper insights into maternal health inequalities.

## 6 Conclusion

This study examined the impact of multidimensional poverty on maternal care utilization in Nigeria, focusing on attendance at eight or more ANC visits and place of delivery. The findings show that non-monetary poverty significantly reduces the likelihood of adequate ANC attendance and facility-based delivery. Other important determinants of maternal healthcare utilization include maternal education, region, place of residence, employment status, health insurance coverage, maternal age, and barriers to accessing care.

## 7 Recommendations

These findings highlight the need for policies that address both monetary (considering household wealth quintiles) and non-monetary dimensions of poverty. Expanding educational opportunities for girls and women, strengthening health insurance coverage for informal workers and rural populations, and improving rural health infrastructure are essential. Mobile health services and community-based interventions can further improve access in underserved areas. Efforts to address sociocultural barriers through gender equity initiatives, male involvement, and community awareness campaigns are also critical. Tackling these structural and social determinants will improve maternal health outcomes and support Nigeria’s progress toward achieving the Sustainable Development Goals.

## Data Availability

The datasets presented in this article are not readily available because the data is hoisted on DHS data website. Requests to access the datasets should be directed to https://dhsprogram.com/data/available-datasets.cfm.
